# STRATEGIES TO ENGAGE LATINOS/X/E IN RESEARCH: CASE STUDIES ON BRAIN HEALTH, PSYCHOSOCIAL WELL-BEING, AND CAREGIVING

**DOI:** 10.1093/geroni/igae098.1406

**Published:** 2024-12-31

**Authors:** Maria Aranda

**Affiliations:** Chair

## Abstract

Several national initiatives underscore the need for innovation in recruitment strategies that diversify community-based research cohorts. In particular, the call for diverse samples, and inclusion of historically underrepresented populations in community-based research is well documented. Prioritizing these goals is paramount, yet challenges remain as to the “how-to” or real-world efforts to advance inclusion of diverse populations in research on aging. This symposium addresses the knowledge gap by drawing from case studies in North Carolina, New York, and Los Angeles, specifically related to the engagement of people of Latino descent. On one hand, we will discuss several core strategies such as fomenting trust, and giving back, which transcend the different studies. On the other, we will highlight different strategies required given the diversity in subgroups, target condition/issue of the study, individual vs. organizational factors, age groups, among others. In our discussion, we will highlight theoretical frameworks such as the Micro-Meso-Macro Framework, Trauma-Informed Principles, and Cultural Adaptation Frameworks. We will conclude with recommendations and future directions for engaging older Latino people in aging research.

